# No-Notice Mystery Patient Drills to Assess Emergency Preparedness for Infectious Diseases at Community Health Centers in New York City, 2015–2016

**DOI:** 10.1007/s10900-018-00595-5

**Published:** 2019-01-02

**Authors:** Mohsin Ali, Marsha D. Williams

**Affiliations:** 1 Bureau of Healthcare System Readiness, Office of Emergency Preparedness and Response, New York City Department of Health and Mental Hygiene, Queens, NY USA; 2 Icahn School of Medicine at Mount Sinai, New York, NY USA; 3 Department of Medicine, Boston Children’s Hospital, 300 Longwood Avenue, Hunnewell 2, Boston, MA 02115 USA; 4 Department of Pediatrics, Boston Medical Center, Boston, MA USA

**Keywords:** Emergency preparedness, Community health centers, Infectious diseases, Drills

## Abstract

Mystery patient drills using simulated patients have been used in hospitals to assess emergency preparedness for infectious diseases, but these drills have seldom been reported in primary care settings. We conducted three rounds of mystery patient drills designed to simulate either influenza-like illness (ILI) or measles at 41 community health centers in New York City from April 2015 through December 2016. Among 50 drills conducted, 49 successfully screened the patient–actor (defined as provision of a mask or referral to the medical team given concern of infection requiring potential isolation), with 35 (70%) drills completing screening without any challenges. In 47 drills, the patient was subsequently isolated (defined as placement in a closed room to limit transmission), with 29 (58%) drills completing isolation without any challenges. Patient–actors simulating ILI were more likely to be masked than those simulating measles (93% vs. 59%, p = 0.007). Median time to screening was 2 min (interquartile range [IQR] 2–6 min) and subsequently to isolation was 1 min (IQR 0–2 min). Approximately 95% of participants reported the drill was realistic and prepared them to deal with the hazards addressed. Qualitative analysis revealed recurring themes for strengths (e.g., established protocols, effective communication) and areas for improvement (e.g., hand hygiene, explaining isolation rationale). We conclude that mystery patient drills are an effective and feasible longitudinal collaboration between health departments and primary care clinics to assess and inform emergency preparedness for infectious diseases.

## Introduction

Recognition of and response to public health emergencies involve many components of the health care delivery system, including primary care clinics. In the primary care setting, infectious diseases pose a particular challenge. Specifically, patients with emerging, re-emerging, or highly infectious diseases may develop nonspecific symptoms (e.g., fever, cough, rash) subacutely. Thus, these patients may be more likely to present to a primary care clinic compared with patients affected by a relatively acute emergency (e.g., natural disasters, fires, radiation, or chemical attacks), who are more likely to present to a hospital emergency room [1, 2]. Hence, primary care clinics are especially important for screening and isolating patients with potentially infectious disease in order to prevent transmission to other patients, health care workers, and the community.

Research on emergency preparedness for infectious diseases has hitherto focused on hospital emergency rooms. Several studies have used “mystery patient drills”—no-notice drills with patient–actors simulating diseases—for avian influenza [3–5], smallpox [6], inhalational anthrax [7], Ebola virus disease [8, 9], Middle East respiratory syndrome coronavirus [10], and measles [10]. These studies enabled assessment of current emergency preparedness protocols, evaluation of the effectiveness of specific procedures within protocols (e.g., use of electronic health record workflows to guide screening), and identification of strengths and areas for improvement. Activities that assess and improve emergency preparedness for infectious diseases in the primary care setting have been relatively underreported, with most published studies either highlighting this as an area of need [11–14] or showing the willingness of primary care practitioners to help in emergency preparedness and response [11, 15].

In order to assess and improve emergency preparedness for infectious disease among primary care clinics, the New York City Department of Health and Mental Hygiene (NYC DOHMH) coordinated a series of no-notice mystery patient drills at community health centers across the city from April 2015 through December 2016. The drills were led by a citywide primary care coalition called the Primary Care Emergency Preparedness Network (PCEPN) [16]. Here we describe both quantitative and qualitative findings of the project and discuss the value of conducting such drills for emergency preparedness for infectious diseases in the primary care setting.

## Methods

### Drill Design and Clinic Selection

The mystery patient drill scenarios were designed by PCEPN with input from their advisory group, which has representation from all five NYC boroughs (Bronx, Brooklyn, Queens, Manhattan, and Staten Island). Scenarios were designed describing a patient walking into a community health center and reporting either (1) fever, generalized body aches, and respiratory symptoms (e.g., cough, shortness of breath) or (2) fever and rash on the trunk and back in the context of attending a children’s birthday party 1–2 weeks prior during which a child may have been sick. These scenarios corresponded to influenza-like illness (ILI) and measles, respectively. Clinics chose their preferred patient scenario in advance of their own drill.

All PCEPN-affiliated community health centers were invited by e-mail to participate; an announcement was also included in PCEPN’s quarterly newsletter. Clinics were enrolled on a first-come, first-served basis, with the number of drills allotted per round dependent on available funding. If a clinic network (defined as the lead institution governing the operation of multiple clinics) participated in multiple rounds, different clinics within the clinic network were prioritized for selection for each round of the drill. The project was exempted by the NYC DOHMH institutional review board.

### Drill Conduct and Evaluation

The drills were completed from April 2015 through December 2016. Drill team staff consisted of an actor, two controllers (one from PCEPN and one from the clinic) who coordinated all drill setup and operations, and one or two trusted agents who acted as planning partners to assist with implementation and execution of the drill at the clinic site. The actor simulating the patient was recruited from the NYC Medical Reserve Corps, a group of volunteer health care professionals trained to respond to public health emergencies. Patient–actors were trained with a 30-min webinar and provided scripts detailing the history of present illness and other relevant components of the medical history. Controllers and trusted agents coordinated, observed, and evaluated the drill. The PCEPN controller directed the drill and accompanied the “patient” as a friend or relative.

To prepare for the drill, all drill team staff completed 3-hour-long webinars (available at https://www.pcepn.org/mpd). In sequence, the webinars (1) introduced the drill and its components; (2) described standard infection control protocols, including considerations for enhancing screening and isolation capabilities; and (3) reviewed the drill plan and logistics. Clinic staff were instructed prior to the exercise to follow their site-specific infection control protocols. Participating clinics that were designated Federally Qualified Health Centers (FQHCs)—organizations receiving grants under United States’ Health Center Program statutes (Section 330 of the Public Health Service Act) [17]—were also provided $1000 USD to purchase supplies or equipment related to emergency management, as an incentive to increase participation. This funding came from NYC DOHMH, supported by Grant 1U90TP000546 from the United States’ Department of Health and Human Services.

Evaluators used a standardized exercise evaluation guide adapted from the Homeland Security Exercise Evaluation Program (HSEEP); the guide included time-based measures for screening and isolation [18]. Time to screening was measured from the moment the patient–actor entered the clinic to the time she or he was given a mask or referred by clinic staff to the medical team because of concern of infection. Time to isolation was measured from when patient was screened to when she or he was placed in a separate and closed room. As a baseline, both time to screening and time to isolation were considered appropriate if performed in under 10 min. The processes of screening and isolation were also graded qualitatively using adapted HSEEP criteria: performed without challenges (P), performed with some challenges (S), performed with major challenges (M), and unable to perform (U). The PCEPN controller concluded the drill when the health care provider began his or her medical evaluation (e.g., taking vital signs, asking the patient’s permission to examine the reported rash). If the patient waited for 15 min without being seen, a trusted agent would inform triage staff to call the “patient,” without disclosing the drill, and proceed with screening to determine if isolation was needed, and, if needed, offer the patient a surgical mask and call a clinical staff member to receive the patient and place him or her in a designated isolation room or space for medical evaluation.

After the drill, the controller and trusted agents debriefed for 30 min with all drill participants (both drill team staff and clinic staff) using the exercise evaluation guide to provide feedback and document strengths and areas of improvement for screening and isolation separately. Each clinic summarized these findings in an after-action report. All drill participants also rated the usefulness of the drill using a 5-point Likert scale.

### Data Analysis

Categorical variables were reported as frequencies and proportions, and continuous variables were reported using median and interquartile range (IQR). Nonparametric methods were used for inferential analyses. In terms of sample size, to detect a change in time to screening or isolation of at least 5 min per round (e.g., 11 min in round 1, 6 min in round 2, and 1 min in round 3), we required 10 clinic networks to participate in multiple rounds of the drill, assuming a two-sided test, alpha of .05, and power of 84% (adjusted from 80% due to 95.5% efficiency of nonparametric tests compared to parametric equivalent) [19]. A p value of < .05 was considered statistically significant, and Stata MP version 14.1 (College Station, TX) was used for data analysis.

For qualitative analysis, after-action reports were reviewed to identify recurring themes (i.e., noted in at least three drills) in strengths and areas for improvement pertaining to screening and isolation.

## Results

Fifty drills at 41 clinics were conducted. In total, 25 clinic networks participated with 14 networks participating in multiple rounds (Table 1). Twenty clinics representing 18 networks participated in round 1 of drills in April 2015; 14 clinics representing 13 networks in round 2 from March through April 2016; and 16 clinics representing 13 networks in the third and final round from November through December 2016. In terms of clinic type, 45 (90%) of the clinics were FQHCs, 4 (8%) were primary care clinics, and 1 (2%) was a FQHC look-alike (a center that fulfills requirements to be a FQHC but is not yet formally certified). Clinics from all five NYC boroughs participated, with 19 in Brooklyn, 16 in Manhattan, 6 in the Bronx, 6 in Queens, and 3 in Staten Island. In terms of drill scenario, 32 were for ILI and 17 were for measles; the scenario for one drill is unknown due to missing data.

**Table 1:** Clinic networks participating in an emergency preparedness mystery patient drill by round, New York City, 2015–2016

Round 1 (April 2015)	Round 2 (March–April 2016)	Round 3 (November–December 2016)
Access CHC Apicha CHC Beacon Christian CHC Bedford Stuyvesant FHC Boriken Health Center Brooklyn Plaza Medical Center CHC of Richmond Community Healthcare Network Harlem United Healthcare Choices Housing Works Joseph P. Addabbo FHC Lutheran FHC (2) Morris Heights Health Center Mount Sinai Beth Israel New York Hospital Queens ODA Primary Health Care Network William F. Ryan CHC (2)	Boriken Health Center Brooklyn Plaza Medical Center Brownsville Multi-Service FHC Community Healthcare Network Damian FHCs Harlem United Housing Works Joseph P. Addabbo FHC Metro CHC Morris Heights Health Center Northwell Health NYC Health + Hospitals (2) ODA Primary Health Care Network	Bedford Stuyvesant FHC Betances Health Center Brooklyn Plaza Medical Center CHC of Richmond Community Healthcare Network Covenant House Harlem United Housing Works Institute for Family Health Joseph P. Addabbo FHC Metro CHC Morris Heights Health Center NYC Health + Hospitals (3) William F. Ryan CHC

If multiple clinics within a clinic network participated within the same round, number of clinics is indicated in parentheses

*CHC* community health center; *FHC* family health center; *NYC* New York City

All but one drill screened the patient within the specified time; in that drill, 18 min elapsed without the patient being called. In the remaining 49 drills, time to screening from entry was known for 45 drills, with a median duration of 2 min (IQR 1–6 min); seven drills (16%) took more than 10 min to screen. HSEEP grading for screening was known for all 50 drills; 35 (70%) of the drills performed the screening without any challenges, 8 (16%) with some challenges, 6 (12%) with major challenges, and 1 (2%) was unable to perform screening.

Masking status was known for 47 drills, of which the patient was masked in 38 (81%) drills. Among the 38 drills in which the patient was masked, 30 (79%) completed this step at the first point of contact in the clinic. Patients presenting with ILI were more likely to be masked compared with patients presenting as measles cases; 28 (93%) of 30 ILI patients were masked compared to 10 (59%) of 17 measles patients (p = 0.007), and 23 (77%) of 30 ILI patients were masked at the first point of contact compared to 7 (41%) of 17 measles patients (p = 0.026). Masking status data were missing from 3 drills.

Among the 49 drills in which the patient was screened, 47 isolated the patient. In the remaining 2 drills, the patient was referred out either to an urgent care clinic or to an emergency room. Time to isolation from screening was known for 41 drills; median time was 1 min (IQR 0–2 min), with no drill taking more than 5 min to isolate. HSEEP grading for isolation was known for all 50 drills; 29 (58%) of the drills performed isolation without any challenges, 17 (34%) with some challenges, 2 (4%) with major challenges, and 2 (4%) were unable to perform isolation.

Analyses for trends across rounds of the drill are shown in Table 2. There were statistically significant trends in the number of patients masked at the first point of contact (47% in round 1 to 75% in round 3; p_trend_ = 0.048) and median time to isolation (0–2 min; p_trend_ = 0.022). There were no statistically significant trends by round in terms of drill scenario (ILI vs. measles), percentage of patients masked, median time to screening, percentage of drills that performed screening without any challenges, or percentage of drills that performed isolation without any challenges.

**Table 2:** Drill scenario and masking, screening, and isolation outcomes, by round of an emergency preparedness mystery patient drill, New York City, 2015–2016

	Round 1 (n = 20)	Round 2 (n = 14)	Round 3 (n = 16)	p_trend_^a^
No. (%) drills with ILI case	11/19 (58%)	8/14 (57%)	13/16 (81%)	.08
Screening				
No. (%) performed without challenges	13 (65%)	10 (71%)	12 (75%)	.26
Median (IQR) mins to screen	5 (1–11)	2 (2–3)	2 (1–6)	.11
No. (%) masked	13/17 (76%)	10/14 (71%)	15/16 (94%)	.11
No. (%) masked by first contact	8/17 (47%)	10/14 (71%)	12/16 (75%)	.048*
Isolation				
No. (%) performed without challenges	10 (50%)	8 (57%)	11 (69%)	.13
Median (IQR) mins to isolate	0 (0–2)	1 (0–2)	2 (1–2.5)	.022*

Denominators are shown for categorical variables for which there was any missing data

*ILI* influenza-like illness, *IQR* interquartile range

^a^P value for trend calculated by Jonckheere’s non-parametric test. Statistically significant indicated by asterisk, using threshold of 0.05

Table 3 shows the median change in duration and quality of screening and isolation per round among 13 clinic networks that participated in multiple rounds of the drill. Six clinic networks participated in all three rounds, whereas seven participated in two of three rounds. There were no significant differences in either duration or quality for either screening or isolation in later rounds.

**Table 3:** Screening and isolation outcomes among 13 clinic networks participating in multiple rounds of an emergency preparedness mystery patient drill, New York City, 2015–2016

	Median (IQR) at initial drill	Median (IQR) change per round	p
Screening			
Time to screen	5 (1–7) min	0 (–2, 0.5) min	.26
HSEEP grade^a^	P (S, P)	0 (0, + 0.5) categories	.46
Isolation			
Time to isolate	2 (0–2) min	0 (–0.5, 0.5) min	.97
HSEEP grade	S (S, P)	0 (0, + 0.5) categories	.10

Six clinic networks participated in all three rounds whereas seven participated in two rounds

p value calculated by Wilcoxon signed-rank test

*HSEEP* Homeland Security Exercise Evaluation Program; *IQR* interquartile range

^a^HSEEP grading categories are: performed without challenges (P); performed with some challenges (S); performed with major challenges (M); and unable to perform (U)

In terms of perceived usefulness, among 132 drill participants from 42 drills representing 22 clinic networks, approximately 95% agreed or strongly agreed to each of the following statements: the drill (1) was plausible and realistic, (2) included participants from appropriate disciplines, (3) engaged participants actively, (4) was appropriate for their level of training and experience, and (5) made them better prepared to deal with the hazards addressed.

Qualitative analysis of after-action reports revealed recurring themes across drills for strengths of both screening and isolation (Table 4); these included presence of established protocols, trained staff, effective communication during the drill, and availability of relevant personal protective equipment and other technical requirements (e.g., masks, alcohol-based hand sanitizer, designated room for isolation).

**Table 4:** Recurring themes for strengths and areas of improvement in screening and isolation identified in after-action reports for an emergency preparedness mystery patient drill, New York City, 2015–2016

	Strengths	Areas for improvement
Screening	Protocols established and staff trained Effective communication PPE available	Masking at first point of contact (clarify indications, e.g., rash) Signage not clearly visible Additional training required Optimized paper/EHR patient registration (to avoid hindering screening)
Isolation	Protocols established and staff trained Effective communication Rooms, PPE, and other requirements identified	Awareness and adherence to requirements (e.g., use of masks) Hand hygiene compliance Explaining rationale of isolation to patient (to minimize psychological impact) Signage for occupied room

*PPE* personal protective equipment; *EHR* electronic health records

In terms of areas for improvement, recurring themes for screening were masking at the first point of contact (e.g., clarifying indications to include a measles-like case of fever and rash without respiratory symptoms), improving visibility of signage instructing patients with pre-specified symptoms and travel history to wear a mask, and optimizing registration procedures on paper-based or electronic medical records such that they do not hinder screening. For isolation, recurring themes for improvement were hand hygiene compliance, awareness and adherence to requirements (e.g., use of masks), explaining isolation protocol to patients to reduce psychological impact of sudden isolation, and using signage to indicate an occupied isolation room.

## Discussion

We conducted 50 mystery patient drills at 41 community health centers across NYC. To our knowledge, this is the largest study of such drills to assess emergency preparedness for infectious diseases in the primary care setting. Only one previous study has reported the use of mystery patient drills for this purpose, with eight drills held in Catalonia, Spain, in 2007, to assess preparedness against avian influenza [5]. In that study, no clinic gave the patient–actor a surgical mask before or after triage, only 2 (25%) performed chest X-rays, and none reported the suspected case to the local public health department. These concerning findings highlight the importance of conducting such drills to assess the emergency preparedness of primary care clinics and identify areas of improvement.

We evaluated preparedness in terms of duration and quality of both screening and isolation. While the median time to screening from entry was 2 min, about one in six drills took more than 10 min to screen the patient. Furthermore, 30% of drills had at least some challenges with screening. After the patient–actor was screened, median time to isolation was only 1 min, with no drill taking longer than 5 min to isolate the patient–actor. However, in terms of quality, 42% of drills had at least some challenges with isolation. Thus, while most clinics screened and isolated efficiently and effectively, there remains substantial room for improvement. In rounds 2 and 3, median time to screening was 2 min compared with 5 min in the first round, though this reduction was not statistically significant. Similarly, there was no statistically significant difference in terms of quality of screening; 65% of drills screened the patient–actor without any challenges in round 1, compared with 75% in round 3. Interestingly, median time to isolation increased in later rounds of the drill, from under 1 min in round 1 to 2 min in round 3 (p_trend_ = 0.022). However, there was no statistically significant trend in terms of quality of isolation; 50% of drills isolated the patient–actor without any challenges in round 1, compared to 69% in round 3. There are multiple explanations for these findings. This study was likely underpowered to detect smaller but still clinically significant differences. While the increase in time to isolation may be due to random error, it may also be due to a clinically significant (but statistically nonsignificant) improvement in quality of isolation. Supporting evidence for the latter explanation is that clinics identified several areas for improvement regarding isolation (e.g., explaining to the patient the rationale for isolation, performing hand hygiene, using signage to indicate occupied rooms), which clinics may have incorporated in later rounds, thereby lengthening the time to isolation.

The patient–actor was appropriately masked in 81% of drills, though this indicates that one in five patient–actors were never masked. Even among those who were masked, one in five were not masked at the first point of contact in the clinic. We also found that actors simulating ILI were much more likely to be masked than those simulating measles (93% vs. 59%, p = 0.007), a finding that persisted when restricting the analysis to only patients who were masked at the first point of contact (77% vs. 41%, p = 0.026). This association may reflect causation since clarifying indications for masking to include a measles-like case of fever and rash without respiratory symptoms was consistently identified as an area for improvement among drill participants.

Furthermore, a greater proportion of cases were masked at the first point of contact in later rounds of the drill (47% in round 1 vs. 75% in round 3, p_trend_ = 0.048), which may reflect improvement by round. However, it may also be due to confounding, as there was a marginally nonsignificant trend for drill scenario by round, with a greater proportion of drills being ILI in later rounds (58% in round 1 to 81% in round 3; p_trend_ = 0.08). Nevertheless, these findings highlight the importance of ensuring masking indications also include potentially infectious patients *without* respiratory symptoms. For measles, this is especially important since it is highly contagious and has recently re-emerged in the face of declining immunization coverage [20]. Newly emerging and re-emerging infectious diseases (a recent notable example includes Ebola virus disease [21]) may also present without respiratory symptoms.

Mystery patient drills for infectious disease of public health concern can serve multiple functions. First, they can provide a practical assessment of emergency preparedness using both quantitative and qualitative methods. Qualitative data are valuable because they can enable triangulation when evaluating quantitative associations, as we show by analyzing participant feedback regarding the exercise that suggests a causal factor to our finding that measles patients were less likely to be masked.

Second, these drills can be used to understand and improve the process of screening and isolation, in part by analyzing components of each process that predict “good performance.” Given the pilot nature of this project, we did not collect quantitative data to analyze components of screening and isolation that were predictive of good performance. However, in our qualitative analysis, drill participants consistently identified use of protocols, appropriate training of staff, effective communication, and availability of required equipment (e.g., personal protective equipment) as strengths in their screening and isolation procedures. Areas for improvement pertained to improving the quality of standard operating procedures, including clarifying indications for masking and isolation, optimizing the registration process to avoid delays in screening, promoting hand hygiene compliance, and explaining to patients the rationale for isolation. This is consistent with a previous study of mystery patient drills for pandemic influenza in the emergency room setting that found that comprehensive standard operating procedures were an important predictor of drill performance [3].

Third, from a public health perspective, mystery patient drills can facilitate an important partnership between community health centers and the local health department for emergency preparedness. In our study, the drills were well received by participants, approximately 95% of whom reported they were plausible, realistic, feasible, engaging, and helped them prepare for the hazards addressed. Thus, these drills can create “buy-in” from clinics and provide useful information to both clinics and health departments. Furthermore, previous research suggests that disaster preparedness drills conducted in community clinics are an effective means to develop and improve clinic emergency plans, specifically regarding when and how to activate such plans, the individual roles of staff, and how to participate in a coordinated response [22].

This study has several limitations. Although it is the largest study to date using mystery patient drills in the primary care setting, the study was likely underpowered to detect smaller but clinically significant differences. This may explain why we found no other statistically significant trends for duration or quality of screening and isolation. Furthermore, as mentioned above, we did not collect quantitative data on components of screening and isolation given the pilot nature of this project. Thus, we could not analyze such factors as predictors of good performance, although we were able to examine factors that were identified in the qualitative portion of our study. Finally, we cannot assess the degree to which desirability bias affected participants’ feedback regarding the usefulness of the drill. However, we encouraged participants to reflect on both strengths and weaknesses of the drill before providing feedback.

Future research using mystery patient drills should aim to further describe and characterize the screening and isolation processes, including analysis of predictors of performance. These drills can also be expanded to evaluate other important outcomes (e.g., decontamination, contacting patient contacts, reporting to the health department). Future studies can also focus on cost considerations and other diseases. Such research will provide insight into the role of mystery patient drills for evaluating emergency preparedness of community health centers and whether these drills can serve as a component of federal certification for these clinics.

In summary, to our knowledge, this is the first study to conduct multiple rounds of drills (three rounds between April 2005 through December 2016) and assess time-based performance metrics (time to screening and time to isolation) in order to investigate the value of these drills in assessing, iteratively informing, and improving the emergency preparedness plans of community health centers. We evaluated screening and isolation of actors simulating patients with ILI or measles in 50 drills at 41 clinics in New York City. In almost every drill, the patient was screened and isolated, although 1 of 3 drills had at least some challenges with either process. Patients simulating ILI were more likely to be masked than those simulating measles, which may reflect a causal association. Approximately 95% of participants reported the drill was realistic and prepared them to deal with the hazards addressed. Qualitative analysis revealed several recurring themes for strengths and areas for improvement. We conclude that mystery patient drills are an effective and feasible longitudinal collaboration between health departments and primary care clinics to assess and inform emergency preparedness for infectious diseases.
